# Bonobos respond aversively to unequal reward distributions

**DOI:** 10.1098/rspb.2024.2873

**Published:** 2025-04-16

**Authors:** Kia Radovanović, Anoek Lorskens, Sebastian Schütte, Juliane Bräuer, Josep Call, Daniel B. M. Haun, Edwin J. C. van Leeuwen

**Affiliations:** ^1^ Department of Biology, Utrecht University, Utrecht, The Netherlands; ^2^ Department of Comparative Cultural Psychology, Max Planck Institute for Evolutionary Anthropology, Leipzig, Germany; ^3^ Department for General Psychology and Cognitive Neuroscience, Friedrich-Schiller-Universitat Jena, Jena, Thüringen, Germany; ^4^ DogStudies, Max Planck Institute of Geoanthropology, Jena, Thüringen, Germany; ^5^ School of Psychology and Neuroscience, University of St Andrews, St Andrews, Fife, UK; ^6^ Global Research Centre for Diverse Intelligences, University of St Andrews, St Andrews, Fife, UK

**Keywords:** bonobo, inequity aversion, cooperation, sociality, comparative psychology, abstract

## Abstract

Inequity aversion (IA) is the resistance to unequitable rewards given similar investments. It has been postulated as an important mechanism by which human cooperation thrives. To understand the evolutionary origin of human IA and its distribution across the animal kingdom, many species have been tested on IA, with mixed results. Whereas chimpanzees were originally found to show IA, more recent studies showed that their IA response could be explained by social disappointment. We conducted two studies on IA in bonobos using established paradigms: a token-exchange task and the social disappointment task. Bonobos could exchange tokens for equal or less-preferred food rewards than their partners (Study 1) and were tested with humans and machines to control for social disappointment effects (Study 2). We found that bonobos responded aversively to unequal food distributions in both studies, which was reflected by more refusals to participate when disadvantaged. Notably, and contrary to chimpanzees, this effect could not be explained by social disappointment, although Study 2 was only partially consistent with an IA explanation. Overall, our findings indicate that bonobos possess the sensitivity to recognize and respond to unfair treatment, which supports the notion that IA may have coevolved as a stabilizing mechanism for cooperation.

## 1. Introduction

Cooperation plays a vital role in humans’ everyday lives, from simple tasks such as sharing household chores to large-scale projects in multi-employee companies. There is even evidence that successful mutual cooperation activates parts of the brain associated with reward processing [1]. However, for cooperation to be evolutionarily stable, the benefits must outweigh the costs [2] and every participant should have consistently engaged in cooperative behaviour during past interactions [3]. This consistency is crucial as it ensures that each group member can expect others to behave cooperatively in future interactions as well, thereby maintaining stability and reliability of cooperation over time. Inequity aversion (IA), which has been defined as a resistance to inequitable outcomes, can serve to prevent free loaders from taking advantage in such situations. Such a resistance is then expressed by individuals relinquishing a material pay-off for the sake of achieving equity [4]. Humans are averse to both disadvantageous inequity, in which they receive the lesser or unfair reward, but also advantageous inequity, where they are the recipient of the better distribution [5]. Recognition of and aversion to disadvantageous inequity can lead to the disadvantaged individual punishing the unfair partner. It also allows them to choose more equitable partners in the future. These actions thereby increase the long-term pay-offs of that individual and, as the unfair individual is excluded from the pay-offs of future cooperative efforts, their fitness is lowered. Seeing an unfair individual be punished may also reinforce more equitable behaviours in others. For advantageous IA, a negative response may signal to future partners that the individual is an equitable cooperator and discourage others from treating them inequitably, increasing their long-term fitness as well [6]. Children already begin paying attention to distributions of payoffs at 1 year old [7] and develop disadvantageous IA in early childhood (advantageous IA is developed in later childhood, but not consistently across cultures [8]). Furthermore, responses can vary according to different factors such as relationship quality, wherein closer relationships may lead to less focus on equity [9] (but see [10] for opposite findings).

Many species of animals participate in cooperative efforts [2] and therefore may also be sensitive to inequity [6]. The evolutionary origins and mechanisms of IA and cooperation in humans can be elucidated by studying them in closely related animal species. The first study on IA in a non-human species was done on capuchin monkeys [11]. Two individual monkeys, the subject and partner, were sat next to each other. In turn, they were given a rock that they could hand back in exchange for a reward. Crucially, in the inequity condition, the partner was rewarded with a high-value reward (grapes) and the subject was given a low-value reward (cucumber) after exchanging. In an equity condition, both monkeys received the same value reward after exchanging. An effort control condition was included, in which the partner did not have to complete a token exchange but received a grape; however, the subject did have to exchange and still only received a cucumber. Additionally, there was a non-social control condition (food control), in which the subject exchanged for a cucumber, but a grape was placed into the empty adjacent room. The monkeys refused to participate significantly more often in the inequity and effort control conditions (but also the food control) compared to the equity condition. These findings led to the conclusion that capuchins are inequity averse.

However, these findings have faced significant scrutiny. For instance, the effect was only found in female capuchin monkeys. Furthermore, the capuchins may have been responding to the presence of the grapes rather than the partner receiving them, as they were not present in the equity condition (also see [12,13]). Another alternative explanation for the capuchins’ refusals was that the capuchins’ expectations of receiving the higher value reward were violated, simply due to seeing their partner receiving it (socially facilitated expectation or the food expectation hypothesis [14]). A third alternative was that a successive negative contrast, i.e. experiencing a downgrade in reward quality due to first being a ‘partner’ (high reward) followed by being the subject (low reward) decreased subjects’ willingness to participate (the frustration effect, also classified under the food expectation hypothesis; see [15]). Some experiments failed to replicate the results found in capuchins [11,13,15–17] (though note that two of these studies did not employ a task). Nevertheless, multiple studies since have incorporated controls for the criticisms mentioned and still found IA to be present [18–20]. Specifically, some studies controlled for the high-value reward presence hypothesis by ensuring the high-value rewards were present in all conditions, regardless of whether anyone received them. For the expectation hypothesis, they created incorrect expectations beforehand or explicitly showed the subject which reward they would receive. Finally, they addressed successive negative contrast by counterbalancing the order of conditions and testing subjects only before they served as partners.

Since the publication of the capuchin study [11], disadvantageous IA has been studied in a wide variety of species, most commonly using variations of the same paradigm (see [21] for a review). Only a few species have shown signs of being inequity averse, even among primate species [22]. However, within the species that have exhibited inequity responses, the common factor among them is that they regularly participate in non-kin-related cooperation [23], supporting the cooperation coevolution hypothesis. A review on fairness responses in primates, however, argued that it cannot be concluded that primates are inequity averse; methodological issues in variations of the exchange paradigm, coupled with findings in alternative fairness studies (e.g. dictator games) point towards primates being rational maximizers instead [24]. Moreover, a recent meta-analysis across 18 animal species found no evidence of IA [25].

In the current study, we focus on responses to unequal reward distributions in bonobos (*Pan paniscus*), one of humans’ closest living evolutionary relatives [26]. Bonobos are a highly social species that continually engages in cooperative activities like mutual grooming, socio-sexual behaviour and intergroup exchanges [27,28]. As such, IA was expected to emerge in the two earlier studies that were conducted in bonobos. In the first study, using a food distribution task while controlling for expectation effects, the researchers found the opposite of an IA effect: the apes (bonobos, chimpanzees, gorillas and orangutans) refused fewer food pieces when the partner received better food [14]. The second study incorporated a token exchange paradigm (deemed necessary to show the IA effect, e.g. see [29]), yet again failed to find indications that great apes (here: bonobos, chimpanzees and orangutans) were inequity averse [20]. The researchers theorized that the lack of replication of results for the apes was most likely due to the elimination of confounding factors, such as order effects and keeping the favoured food visible in all conditions [14,29].

However, these two studies in which no IA effect was found analysed all great ape species together [14,20]. When scrutinized per species, the respective studies seem to suggest that bonobos responded much more strongly to unequal reward distributions than chimpanzees (e.g. see fig. 1 in [20]). More recently, a third study has been conducted in which bonobos indeed seem to respond aversively to unequal reward distributions [30]. In this study, bonobos were tested in a token exchange task where they were disadvantaged, equally, or better rewarded than their partner, yielding bonobos to refuse more exchanges and rewards when they received the less preferred reward. However, this study was unable to rule out important alternative hypotheses that have been identified in the study of IA in animals such as the food expectation [13,14] or the social disappointment [31,32] hypothesis. According to the disappointment hypothesis, subjects expressed their disappointment in the human experimenter for not giving them the more preferred food.

Engelmann *et al.* [31] were the first to create an exchange paradigm controlling for the social disappointment hypothesis. Chimpanzees were put into a token exchange paradigm where the rewards would either be distributed by a machine or by a human, and either in the presence of a partner or alone. In this study, no equity condition was included, but instead, across all conditions, the subjects received a lower value reward while the partner always received a higher value one. For the subject, an opt-out option was also included, through which it could reject the given reward without sacrificing resources. The food expectation hypothesis predicted that negative reactions would be the same in all conditions. The social disappointment hypothesis predicted that negative reactions would be highest in both conditions where the rewards were distributed by a human. The IA hypothesis predicted negative reactions would be highest in the two conditions where the subject had a partner. The chimpanzees refused to exchange more when the distribution was offered by a human rather than by the machine, regardless of whether there was a partner present, supporting that they are sensitive to the actions of a human rather than inequity *per se* [31]. A recent study investigating social disappointment in long-tailed macaques found similar results [32], but to our knowledge no other studies have tested the social disappointment hypothesis.

Here, we set out to test bonobos with two established paradigms that can rule out alternative explanations. In our first experiment, we replicated the study by Bräuer *et al*. [20] in which great apes exchanged tokens for rewards that were either equal or of lesser value to their partners’ rewards. This study controlled for the alternative effects of the presence of the high-value food and the order effects of testing. Here, we hypothesized that bonobos would show IA by refusing to participate more frequently in the unequal compared to the equal condition. To corroborate our efforts, we additionally re-analysed the data from the original Bräuer *et al*. [20] study and checked for consistency in findings as well as for an overall effect of inequity on bonobos’ responses by collating the two datasets. In our second experiment, we replicated the study by Engelmann *et al.* [31] in which chimpanzees were shown to respond based on social disappointment motivations rather than IA. Their study was the first to implement a condition in which an automated apparatus distributed the rewards rather than a human experimenter, which tested the alternative hypothesis that chimpanzees were expressing their disappointment in the experimenter for not giving them the more preferred reward when they could have. Moreover, a so-called ‘opt-out’ apparatus was also available to the subject; this apparatus was separate from the main exchange one and was baited with the less preferred food by a different experimenter than the one distributing the rewards during the trials. Therefore, the opt-out represented a non-costly option for the bonobo to reject the reward distribution relative to the partner but still obtain a food reward. In addition to the distributor type condition, there was a non-social control condition for both types of distributors.

We tested three hypotheses: (i) food expectation—negative responses are due to the expectation that they will receive the preferred food based on past experience of themselves or their partner receiving it; (ii) social disappointment—bonobos are expressing their disappointment in the experimenter by refusing to exchange; and (iii) IA—negative responses are a reaction to the perceived inequity between the effort and payoff of the subject and partner. Hypothesis (i) predicts no significant differences in negative reactions between the four conditions; hypothesis (ii) predicts the negative reactions to be higher in the two human distributor conditions compared to the machine distributor ones; and hypothesis (iii) predicts the negative reactions to be higher in the two partner conditions compared to the non-social ones. Given the precedent in the human [9] and other animal literature on IA (chimpanzees [33]; bonobos [30]), we further investigated whether affiliation (also known as ‘friendship’ in the human literature) impacted the degree of tolerance to inequity. We hypothesized that IA would become less pronounced with increasing levels of dyadic affiliation. In addition, we considered how these findings contribute to understanding the evolutionary development of IA, including whether it arose through convergent evolution as a response to similar social pressures across species.

## 2. Material and methods

### (a) Subjects

Six bonobos (three male and three female) participated in the studies. Their ages ranged from 9 to 39 years old. Two of them were hand-reared and four of them were mother-reared (see electronic supplementary material, table S1). The bonobos were housed in one social group at the Wolfgang Kohler Primate Research Center in the Leipzig Zoo (Germany). Their housing consists of an indoor enclosure and sleeping room, whereas the outdoor enclosure was closed at the time due to cold weather. The bonobos regularly participate in cognitive testing and were already trained by their keepers to hand back items in return for a reward. They receive regular feedings and enrichment, and have water available ad libitum. At no point were the bonobos deprived of food or water and they participated in the study voluntarily.

### (b) Ethical assessment

The studies were approved by the ethics committee of the Max Planck Institute for Evolutionary Anthropology and Leipzig Zoo. No medical, toxicological or neurobiological research of any kind is conducted at the WKPRC. All research strictly adheres to the legal requirements of Germany. Animal husbandry and research at the WKPRC comply with the ‘EAZA Minimum Standards for the Accommodation and Care of Animals in Zoos and Aquaria’, the ‘WAZA Ethical Guidelines for the Conduct of Research on Animals by Zoos and Aquariums’ and the ‘Guidelines for the Treatment of Animals in Behavioral Research and Teaching’ of the Association for the Study of Animal Behavior (ASAB).

### (c) Study 1—token exchange paradigm

Study 1 replicated an established token-exchange task in which individuals hand back a token in return for a food reward. Using a within-subjects approach, two conditions are tested: an *equity* condition in which the partner receives the same low-quality food reward as the subject, and an *inequity* condition in which the partner receives a high-value reward while the subject receives a low-quality reward [20]. To establish which were the low and high-quality rewards, a food preference test was done prior to testing (see electronic supplementary materials for detailed procedure).

We followed the procedure as outlined in the original study [20]. The subject was tested with a partner in an adjacent room in which they could see each other. Both were alone in their rooms other than in the case of mothers with offspring. The experimenter sat in the booth between the two rooms. The two buckets containing the low- and high-value reward were placed in a line in front of the experimenter, equidistant from the rooms, so that the bonobos could always see inside them.

Testing began when the experimenter handed the partner bonobo the token (a 20 cm × 4 cm plastic tube). To signal that the token should be returned to the experimenter, they held their hand open in front of the mesh openings. If the partner gave it back within 60 s, the experimenter took the token and returned the appropriate reward (carrot/apple in the *equity* condition, grape in the *inequity* condition). This same procedure was subsequently done with the subject, except in both conditions the subject was given the carrot/apple. The exchange with both was considered to be one trial.

If the bonobo did not take the token, the experimenter kept calling their name and gently rattled the token in the openings to try and get their attention. If they still did not take it within 60 s, the experimenter moved on to the other bonobo. If the bonobo did not return the token immediately after taking it, the experimenter kept signalling they wanted it back by opening and closing their hand or further calling the bonobo’s name. Again, if they did not return the token within 60 s, the experimenter moved on to the other bonobo.

Each session consisted of 25 trials. A bonobo could only participate in one session per day as a subject but could participate in another one on the same day as a partner in a different dyad. All possible combinations of dyads were tested. In the *inequity* condition, only the bonobo receiving the lower value reward was a subject, whereas in the equity condition, both received the same. Hence, each dyad was tested twice in the *inequity* condition, switching between who served as subject and who as partner. In the equity conditions, both received the same reward. Therefore, only one equity test per dyad was needed, where both bonobos served as subjects. The order of who began the token exchange first was random for the equity conditions. The order of testing the three sessions for each dyad was randomized (electronic supplementary material, table S2).

Coding was done as in Bräuer *et al*. [20]. We coded refusals as not returning the token within 60 s after receiving it or not consuming their food within 5 s of receiving it. Out of 1138 trials, refusals occurred in 57 of them and only 2 of those were food refusals. Successful exchanges were coded when the bonobo handed back the token within 5 s of receiving it, which occurred 914 times. Exchanges between 5 and 60 s were not included in the statistical analyses. In some trials, the experimenter did not exactly replicate the original procedure by Bräuer *et al*. [20] of offering the token even if the bonobo wasn’t in front of them, but rather waited for them to come back to have their full attention, or offered it, but only counted the beginning of the trial from when the bonobo came to take it. To obtain an accurate replication of the original study, we have therefore discarded the trials (*n* = 60) with incorrect procedure and recoded those where their timing for coding was incorrect. All coding was done from video recorded during the experiment. A second rater coded 15% of the videos to reach an interrater reliability with the first coder of 100%. To boost sample size and hence the power of the current study, we combined our data with the data on bonobos from the original study [20]. Given that the two studies were replicates, this was possible (see *Statistical Analysis*).

### (d) Study 2—social disappointment paradigm

Study 2 replicated the social disappointment study in chimpanzees in which it was tested (and found) if the observed IA effect in chimpanzees could alternatively be explained by a sense of disappointment in the human experimenter when they did not provide the same high-value reward to the subject as they did to the partner [31].

We followed the procedure as outlined in the original study (see [31] and electronic supplementary material for details). In summary, the bonobos were again tested in pairs, where the subject was always confronted with an unequal food reward distribution (food value was assigned according to the same food preference test as in Study 1). However, there was a scenario in which either a human experimenter or an automated machine administered the food rewards, in either the presence of the partner or alone. Thus, a 2 × 2 design was used, with *distributor* (human or machine) as a between-subjects variable and *partner presence* (yes/no) as a within-subjects variable.

Importantly, subjects were always tested in unequal (disadvantageous) reward distribution conditions. The experimenter (E1) or the machine provided the subject with a lower food reward than the partner or empty room. Each subject was tested in four testing sessions of 24 trials each, with only one session per day. Due to zoo protocols limiting the length of testing time per day, sometimes sessions could not be completed within one day and were then continued another day to reach 24 trials. Two of the four sessions were with no partner consecutively and the other two were with a partner consecutively. Which subjects started their first trial with or without a partner was counterbalanced (electronic supplementary material, table S3, it is not fully balanced but we account for this in our statistics). Within all four sessions, the subject remained in either the human or machine distribution condition. Before the beginning of each trial, an experimenter (a different one than the one doing the food distribution in the human condition; E2) entered the testing room to reset and rebait the apparatuses. Once the apparatuses were set and baited, E2 left the testing room and either E1 entered and made the token available to the bonobo by hand (human condition) or E2 left the room and the machine ‘itself’ made the token available to the bonobo (machine condition; this was achieved by the experimenter pulling a string attached to the machine from outside the testing room). The trial ended once the subject had reinserted the tool either into the main apparatus or the opt-out, had refused to exchange the tool within 30 s of receiving it, or had refused to take the tool within 30 s of it being made available.

We must note that in the machine/alone condition, we originally ran the tests without being able to make the rewards drop down into their final distribution for the partner apparatus, as there was no bonobo to trigger the apparatus to do so. Without this step, the subject could not see which of the two rewards would be offered to the empty space the partner was in in the other half of testing. Therefore, we readjusted the apparatus and re-tested this condition with the three subjects who were assigned to the machine distributor condition. For the results obtained with the original data, see the electronic supplementary materials.

Coding was done as per Engelmann *et al.* [31]. The dependent variables measured were refusal to initiate, refusal to exchange or consume food, choice of opt-out, and successful exchange with main apparatus. Refusal to initiate was coded if the subjects did not take the tool out of the apparatus within 30 s of it being made available to them. Out of a total 571 trials, refusals to initiate occurred in 70 of them and were excluded from the statistical analyses. Refusals to initiate were excluded as the subjects at this point still did not know which of the two rewards they would be receiving. Therefore, a refusal of inequity is not possible as they did not know it existed yet [31]. Refusal to exchange was coded when the subject did not reinsert the tool into either the main or opt-out apparatus within 30 s of removing it. Food refusals were coded if the subject did not consume the food within 30 s. Refusals to exchange or to consume the food were counted together and occurred 45 times, only 1 of which was a food refusal. Choice of opt-out was coded when the bonobo inserted the tool into the opt-out apparatus within 30 s of obtaining it, which happened in 112 trials. Successful exchange was coded when the bonobo inserted the tool into the main apparatus and obtained the reward within 30 s of obtaining the tool. All coding was done from video recorded during the experiment. A second rater coded 15% of the videos to reach an interrater reliability with the first coder of 100%. During coding, we also noted that in two of the trials of a mother who could not be separated from her dependent offspring, her son took the tool out himself when it became available. The mother, our subject, took the tool from him as soon as she realized and proceeded as she would normally. Therefore, we included these trials in our data and counted initiation of the trial as the moment she took the tool from her son.

Lastly, to operationalize affiliative relationships, we conducted group scans [34]. When the bonobos were freely roaming in their indoor enclosure, every 10 min, we scored proximity and grooming. Proximity was coded as being within an arm’s length from other individuals; grooming was coded when individuals engaged in going through the fur of another individual using their fingers (for more details, see electronic supplementary materials).

### (e) Statistical analysis

We used generalized linear mixed models (GLMMs) with binomial error distribution and logit link function to model the probability of refusals in both studies. All models were fitted in R (version 4.2.1 [35]) using the package lme4 and the function glmer [36]. First, when testing multiple effects at once, full-null model comparisons were conducted to test the overall fit of the full model and avoid ‘cryptic multiple testing’ using likelihood ratio tests (LRTs) [37]. If the full-null model comparison indicated a significantly better fit of the full model to the data, then parameter estimates were derived using LRTs with the ‘drop1’ function from the lme4 package. Confidence intervals were obtained with the confint.merMod function (Wald method) of the lme4 package. Contrasts between levels of factors were assessed with the emmeans package, which adjusts the respective *p*-values using the tukey method for comparing a family of n estimates [38]. *p*-values < 0.05 were considered significant.

#### (i) Study 1

Here, we replicated the study by Bräuer *et al*. [20]. We investigated whether bonobos were more likely to exchange tokens when their partner received the same (equity) versus a better (inequity) food reward. The response variable was yes/no token exchange (binomial error distribution) in a given trial (‘no’ equates to refusal). In the first model, we tested a main effect model only, meaning that the only fixed effect we included was ‘condition’ (inequity/equity). This model thus equates to a full-null model comparison. We included the random intercepts of both bonobos’ identities (subject, partner) including their unique combination (dyad) to account for repeated testing. In this model, to boost power, we collated the data from the original study by Bräuer *et al*. [20] with our data resulting in an effective sample size of 9 subjects, 46 unique dyads and 2027 trials. Two of the nine subjects were tested in both studies (see electronic supplementary material, table S1), which was accounted for by the random effects structure [39]. Due to the fact that the original data came without details on trial, session and the order by which condition was administered, here we focus on the main effect of ‘condition’ and investigate in several ways if the effect is robust: (i) we tested whether the data from the original study differed from the current study by adding an interaction between condition and study, which was not significant, indicating that both studies yielded the same effect (LRT: *χ*^2^ = 0.91, df = 1, *p* = 0.34); (ii) we performed a model stability check in which we re-ran the model with each time a single dyad removed (with replacement) to assess the impact of certain individuals and dyads on the ‘condition’ estimate; and (iii) we ran a data simulation to estimate the power of our model to detect a ‘condition’ effect by simulating responses in the context of our model (thus including the small sample size) and checking to what extent our model was able to retrieve the original estimate (script available upon request).

In a second model, we investigated whether the probability to exchange was moderated by dyadic relationship quality in the form of proximity and grooming indices. Here, we were not able to combine the datasets because proximity and grooming measures were not obtained during the original study period [20]. This single-study focus allowed us also to model the impact of trial (z-transformed), session (z-transformed) and order of condition administering, as we did collect these meta-data for the new sample. To test the moderation effect of relationship quality, we tested the interaction of both the social indices with ‘condition’. We also included ‘sex’ (of subject) to match the analysis of Study 2 (see below), and the interaction between ‘order’ of testing and ‘condition’ to test if refusals were more frequent when subjects were tested in the equity condition first (full model). As null model, we used the model with trial, session, sex and order of testing (thus omitting the focus variables ‘condition’, ‘grooming’ and ‘proximity’, including their interaction). The random effects structure of both the full and null model consisted of the intercepts for subject, partner and dyad, as well as the respective slopes for trial and session [39]. The sample for this model included 6 subjects, 26 unique dyads and 1027 trials. The full model provided a slightly better fit to the data than the null model (*χ*^2^ = 10.78, df = 6, *p* = 0.095), which we took as justification to explore the effects further (see §3).

#### (ii) Study 2

Here, we replicated the study by [31]. In the first model, we investigated whether bonobos were more likely to exchange tokens when the inequity was created in the absence versus the presence of a partner, and whether this differed when the inequity was created by a human versus a machine. The response variable was yes/no exchange (Bernoulli distribution) in a given trial (‘no’ equates to refusal). Trial (z-transformed), session (z-transformed) and sex were included as fixed control effects in the model (null model), as well as the two-way interaction between distributor (human or machine) and partner presence (yes/no), which was included in the full model. To control for potential effects of the bonobos having their first sessions with or without a partner, we also included order as a fixed effect into the null model. Furthermore, we included random slopes of partner presence, session, and trial within both tested bonobos (intercepts), and session and trial within dyad (intercept). The sample for this model included 6 subjects, 16 unique dyads and 501 trials. The full model provided a better fit to the data than the null model (*χ*^2^ = 16.55, df = 3, *p* < 0.001). As in Study 1, here we tested for the stability of the results by performing model stability checks and running data simulations (see (i) Study 1).

Similar to Study 1, in a second model (with only the ‘partner present’ trials), we included the relationship quality variables proximity and grooming. The sample for this model included 6 subjects, 16 unique dyads and 247 trials. The full model provided no better fit to the data than the null model (*χ*^2^ = 0.96, df = 2, *p* = 0.618).

In a third model, the response variable was yes/no opt-out use (Bernoulli distribution) in a given trial (‘no’ means they did not use the opt-out). The fixed effects and random effects were the same as in the first model. The full model provided no better fit to the data than the null model (*χ*^2^ = 2.16, df = 3, *p* = 0.54).

## 3. Results

### (a) Study 1

**Figure 1.**
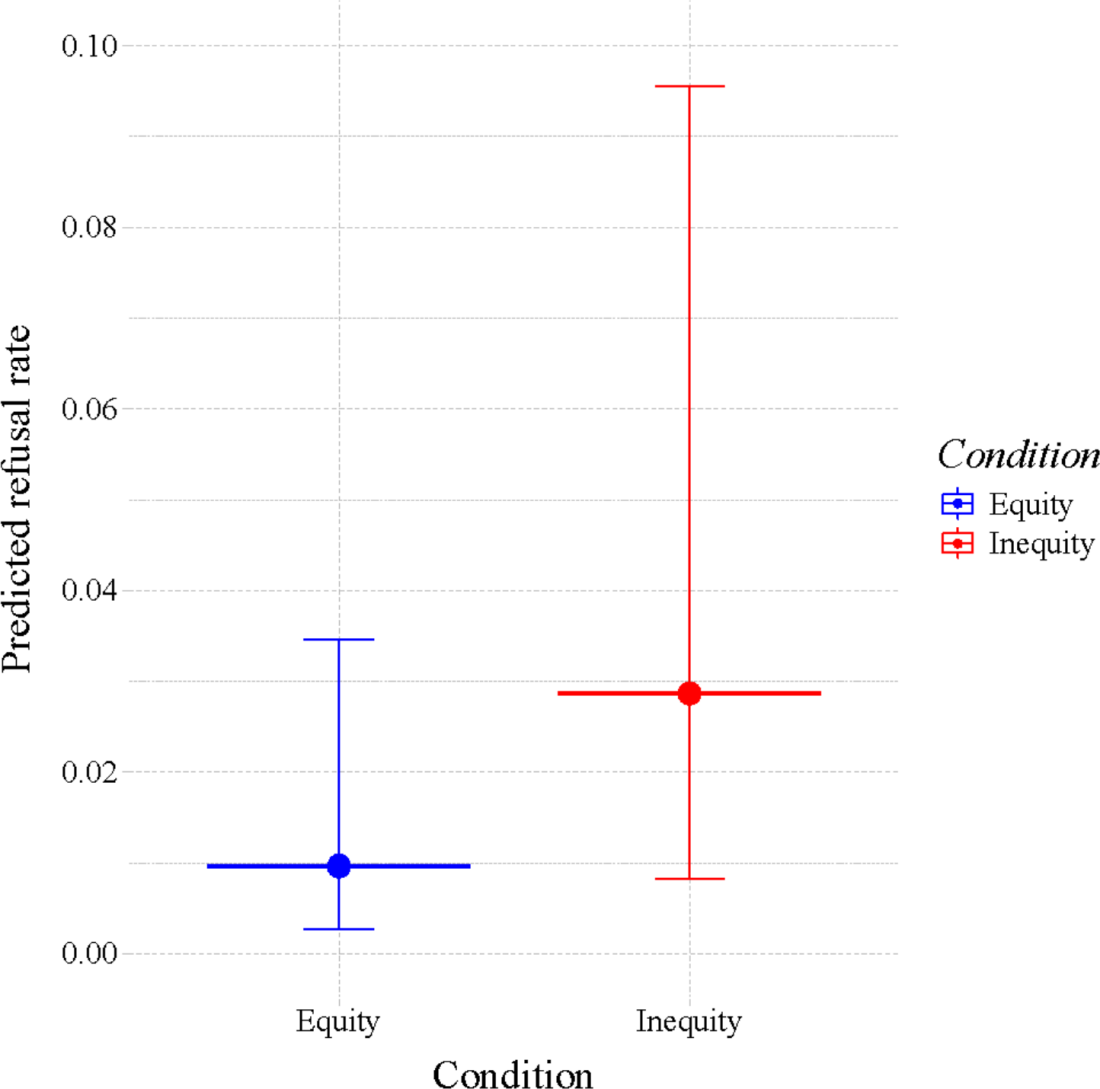
Bonobos refuse to exchange (*y*-axis) more when their partner receives better food rewards (inequity condition; *x*-axis), although this result was unstable (see (a) Study 1). Depicted are the model-derived predicted values, including their 95% CIs (vertical lines extending from the large dots representing the mean).

Bonobos were significantly less likely to exchange tokens with the experimenter when the partner received better food rewards compared to when the partner received the same food reward as the subject (LRT, within-subjects main effect ‘condition’: χ^2^ = 37.78, df = 1, *p* < 0.001, *n* = 9; figure 1). In the equity condition, the odds for subjects to exchange tokens were exp(1.18) = 3.25 times larger than in the inequity condition (estimate ± SEM = 1.18 ± 0.20: 95% CI = 0.79–1.56).

We chose to test the condition effect in the strongest possible way, which is by combining the datasets. In this context, the interaction between condition and study is not significant (*p* = 0.34; see §2e), and the separate main effects are significant (original study [20]: *χ*^2^ = 34.74, df = 1, *p* < 0.001; our study: *χ*^2^ = 7.06, df = 1, *p* = 0.008).

Inspection of model stability metrics indicated that the model was stable—the ‘condition’ effect was robust against the sequential omission (with replacement) of dyads (electronic supplementary material, figure S1). Data simulations additionally showed that the ‘condition’ effect could be readily identified by the model, which had a power of 0.98 for retrieving the original condition estimate (electronic supplementary material, figure S1).

**Figure 2.**
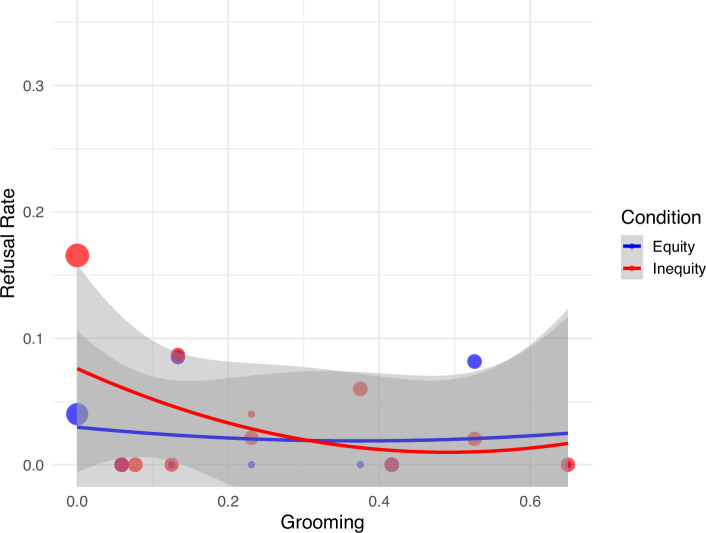
Bonobos’ refusal to exchange (*y*-axis) decreases steeper with increasing measures of social bonding in terms of grooming relationship quality (*x*-axis) in the condition where the rewards were distributed unequally (partner receiving better food reward). Depicted are the actual data (dots) including their model-derived trend lines.

Relationship quality affected the probability of refusing to exchange in each condition differently. With increased levels of grooming between the subject and partner, the subject’s probability to refuse to exchange tokens decreased more in the inequity condition than the equity one (LRT interaction between ‘grooming’ and ‘condition’: *χ*^2^ = 4.48, df = 1, *p* = 0.034; figure 2).

For every 1-unit increase in standardized grooming (z.grooming), the log-odds of refusal in the inequity condition decrease by 1.29 units more strongly than in the equity condition. In other words, this indicates that the odds of refusal associated with increased grooming in the inequity condition are approximately exp(−1.29) = 27.6% of those in the equity condition for the same level of grooming. However, this was only the case for relationship quality expressed in terms of social grooming, not for proximity (LRT: *χ*^2^ = 1.14, df = 1, *p* = 0.28). Moreover, inspection of the model stability and data simulation results indicate that the interaction effect was relatively unstable (electronic supplementary material, figure S3), with the model only reaching a power of 0.281 to retrieve the significant effect of the interaction between grooming and condition. Thus, the results with respect to the influence of affiliation on bonobos’ responses to inequity should be considered with caution.

Lastly, we found no significant effect of the order by which the conditions were presented to the bonobos (LRT ‘order’: *χ*^2^ = 2.13, df = 1, *p* = 0.15), nor for trial number (*χ*^2^ = 2.44, df = 1, *p* = 0.12), session (*χ*^2^ = 0.46, df = 1, *p* = 0.49) or sex (*χ*^2^ = 0.27, df = 1, *p* = 0.60).

### (b) Study 2

**Figure 3.**
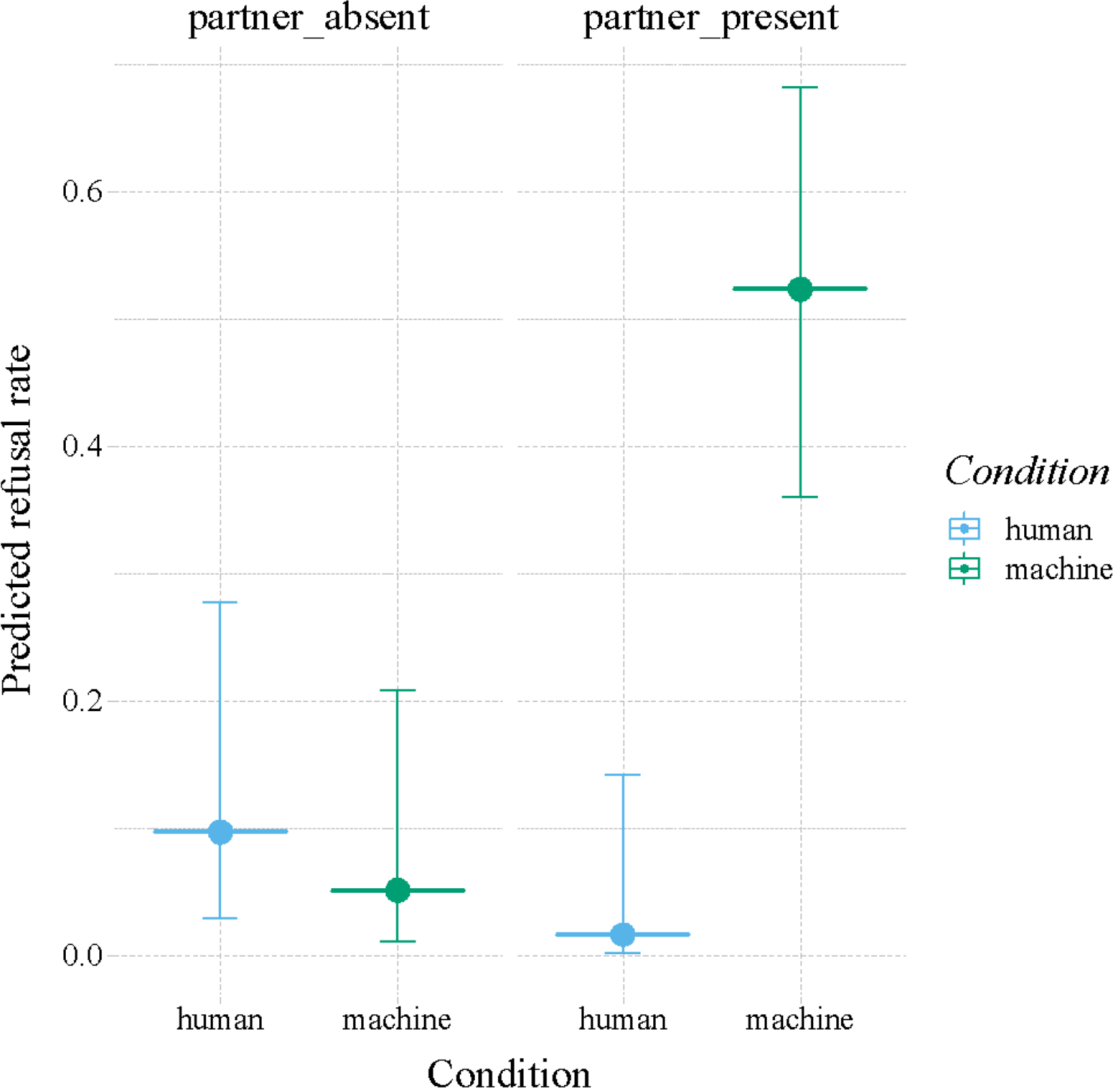
Bonobos refuse to participate most frequently in the condition where the rewards are distributed by the machine (green) and the partner is present (right) to benefit from the (better) reward. Depicted are the model predictions for refusal rates (*y*-axis) for all conditions (*x*-axis). Circles represent the point estimates, the extending vertical lines represent the 95% confidence intervals.

The probability for bonobos to exchange tokens was dependent on whether a human or machine was distributing the rewards moderated by whether or not a partner was present (LRT interaction distributor|partner present: *χ*^2^ = 9.31, df = 1, *p* < 0.003). Specifically, the bonobos refused most frequently in the condition where the rewards were distributed by the machine and the partner was present to benefit from the better reward (figure 3). In this condition, the bonobos were more likely to refuse than in the ‘machine|no partner’ condition (estimate ± s.e. = 2.97 ± 0.83, *p* = 0.002), the ‘human|partner’ condition (Estimate ± s.e. = 4.19 ± 1.23, *p* = 0.004), and the ‘human|no partner’ condition (estimate ± s.e. = 2.32 ± 0.76, *p* = 0.012; see figure 3).

Inspection of model stability metrics indicated that the model was stable—the interaction effect of distributor and partner presence was robust against the sequential omission (with replacement) of dyads (electronic supplementary material, figure S4). Data simulations additionally showed that the ‘distributor|partner’ interaction effect could be readily identified by the model, which had a power of 0.824 for retrieving the original estimate (electronic supplementary material, figure S5).

Bonobos’ refusal to engage in the token exchange was not moderated by levels of affiliation in terms of proximity and grooming (i.e. the full model did not provide a better fit to the data than the null model, see §2). Furthermore, we found that the bonobos were more likely to refuse across sessions (*χ*^2^ = 4.45, df = 1, *p* = 0.035; estimate ± s.e. = 0.93 ± 0.44) and when they first experienced a condition *without* a partner (*χ*^2^ = 4.26, df = 1, *p* = 0.04; estimate ± s.e. = −2.32 ± 1.26). We found no statistically significant effects for sex (*χ*^2^ = 0.15, df = 1, *p* = 0.70) or trial number (*χ*^2^ = 2.63, df = 1, *p* = 0.11).

## 4. Discussion

Bonobos responded negatively to unequal reward distributions in two experimental paradigms by refusing to exchange tokens more frequently when they were disadvantaged in terms of the relative reward quality that they received. In both paradigms, the apes were required to put effort into the experiment, which has been established as a prerequisite to elicit IA in other species (e.g. see [18]). Moreover, their reluctance to exchange tokens when faced with unequal reward distributions could not be explained by the social disappointment effect, which has recently been identified as an alternative explanation for earlier findings on IA in chimpanzees [31].

Our first study, in which the bonobos refused to exchange the token more often when they were unequally versus equally rewarded to their partner, was a replication of an earlier study on IA in bonobos [20]. In the context of replication, we identified a consistent pattern between the original and our replicated findings with bonobos refusing more in the inequity compared to the equity condition. The protocol of the original study [20] was an improved version of previous studies on IA in primates (e.g. capuchin monkeys [11]) in that the paradigm was able to control for the presence of the preferred food and order effects of testing. This was done by having both types of food visible in both the equity and inequity conditions and by randomizing the order of the conditions tested within each dyad, respectively. This original study [20] tested three species (bonobos, chimpanzees, and orangutans), which, pooled together, showed no evidence of IA, yet showed that bonobos exhibited the highest rate of refusals ([20] figure 1). This finding is supported by the recent evidence of Verspeek & Stevens [30], who also found indications that bonobos respond aversively to inequitable reward distributions [30]. Yet, importantly, the methodologies in both these studies did not control for the social disappointment hypothesis or the expectation of receiving the food caused by moving the preferred food in the presence of the subject (i.e. there was no non-social control). These important caveats were addressed by our second study.

In our second study, we used the paradigm that has recently been used to show that chimpanzees are not necessarily inequity averse, but are instead sensitive to the actions of the human experimenter [31]. In contrast, in the current study, bonobos most frequently refused when they were disadvantaged and paired with a partner who received a reward from the machine. This finding challenges the idea that their refusals are driven by social disappointment. Interestingly, however, while the bonobos showed higher refusal rates in the inequity condition when rewards came from the machine, they did not exhibit a similar increase in refusals in the human condition, which is both intriguing and difficult to interpret. This is especially so in light of the results from Study 1, in which the bonobos refused to exchange more often when confronted with an unequal reward distribution presented by a human experimenter, leading one to expect the same outcome in the human condition of Study 2. We do not currently have a plausible explanation as to why this lack of refusals occurred in the human condition. We speculate that chimpanzees and bonobos may engage in different interactions with the experimenters, where chimpanzees may respond under the assumption that the experimenters may change their behaviour (despite them being consistent in providing the same quality rewards across conditions, contrary to Study 1), while the bonobos may not be triggered by this abstract possibility (cf. Study 1, in which the bonobos experience the distinction in reward quality first hand), and/or adhere more docile to the experimenters’ provisioning decisions. A research design to explore this possibility could incorporate humans preparing the rewards yet leaving the room before the bonobos actually receive it. For now, given the consistency of results of Study 1 and the machine condition of Study 2, we cautiously interpret the bonobos’ responses as another empirical argument in favour of IA in bonobos (also see [30]), while stressing the need for further research.

Bonobos have been reported to be more tolerant than chimpanzees, possibly leading to more effective cooperation strategies [40] (but see [41] for some nuance)—this difference in social propensities may mean that bonobos have a higher sensitivity to inequity than chimpanzees, which could account for the opposing findings. Alternatively, these opposing findings may be circumstantial, and both bonobos and chimpanzees may (sometimes) behave aversively towards inequity, dependent on group dynamics. Although the studies that have reported IA in chimpanzees (e.g. [18,42]) may reflect that chimpanzees can be sensitive to inequity, the lack of relevant control conditions in those studies dictates caution in interpreting those results. Based on the theorized link between cooperation and IA [22,43], it is conceivable they are sensitive to unfairness given that chimpanzees are known to engage in cooperation frequently and in diverse contexts [44,45].

Interestingly, in Study 1, we also found that closely affiliated dyads were more tolerant of inequity, as they refused to exchange less in the inequity condition when paired with more closely bonded partners (Study 2 did not show any effect of affiliation, likely due to a lack of power). Humans report using communal need-based principles for the distribution of benefits with those they have close relationships with, while resorting to distributive justice principles of equity with those they do not [9]. However, there is also experimental evidence showing that humans have a greater dislike for unfair offers from friends than strangers [10]. In line with this, male marmosets only display IA with their pair-bonded mates [46]. In our paradigm, the subject was not receiving an offer from the partner but the experimenter. Despite this, the effect of partner affiliation in Study 1 suggests that the subjects might be responding to the perceived unfairness of their partner receiving a different reward. Specifically, they may be more tolerant of their partner receiving an unequal reward when that partner is a close friend, regardless of who is offering it.

Notably, we measured affiliation in two different ways: grooming and proximity rates. The effect of affiliation was found only with the grooming network, not with proximity. This finding supports the idea that different methods of measuring affiliation may reflect different aspects of relationships [47], which in turn may explain the conflicting results within and across species. In an IA study with dogs, when affiliation was measured by whether they slept with body contact, the dogs required more commands to complete the task when paired with conspecifics they were highly affiliated with; when affiliation was measured by food tolerance in the same study, there was no correlation to negative responses to inequity [48]. Additionally, with chimpanzees, the quality of relationships, based on a weighted average of grooming, contact and proximity, did not correlate with refusals to exchange. Nonetheless, pairs that had lived together longer were less likely to refuse in the inequity condition [33,49].

Humans, chimpanzees and bonobos are closely related, so similar behavioural patterns might be expected across these species. However, bonobos exhibit distinct tendencies. Unlike humans, who favour familiar individuals, bonobos show an attention bias towards strangers [50,51]. They also share food with unfamiliar individuals, unlike chimpanzees [52]. Based on these behaviours, it might be expected that bonobos would tolerate inequity more from strangers. Yet, our findings suggest otherwise. On the other hand, as our findings were based on an unstable statistical model, further research is needed to clarify the role of affiliation in IA.

There were a few limitations in our study. The first is the fact that due to having to re-test the machine-alone condition, all subjects started with the partner-present trials in the machine condition, which exhibited the most elevated refusals. The lack of counterbalancing warrants caution when interpreting the results, though it should be noted that the data from the incomplete testing trials produced very similar results as the repeated tests that were executed completely. We also had a small sample size, which is a common issue with experimentally testing captive primate groups. We attempted to alleviate this issue as much as possible by combining our new dataset with the original one from Bräuer *et al*. [20], and assessing the power of our models with post-hoc data simulations. Yet, testing multiple groups of bonobos is important both to increase sample size and to account for the varying responses given by different groups and populations [53–55]. Furthermore, in Study 2, due to time and logistic constraints during testing, we could not keep the partners for each subject consistent. Consistency would have been better for the sake of more accurate replication of the original study [31]. Moreover, the greater mix of partners may have obscured an effect of affiliation on refusals, as there were much fewer trials per partner to be analysed compared to Study 1. Lastly, to assess the form and function of IA more naturalistically, a paradigm in which dyads directly cooperate with each other may be more valid. Similarly, the current exchange paradigms do not allow the subjects to change their situation, rendering their refusals somewhat futile (also see [24]). If the subjects indeed perceive the futility of their actions, this limitation may partly explain the relatively low number of refusals throughout both studies. This issue could be solved by ultimatum games in which reward distributions can be accepted or refused by the subject (e.g. [56]), or by paradigms in which the subjects can actively alleviate their disadvantaged predicament [57]. Therefore, the findings, while partially suggestive of IA, must be interpreted cautiously due to the above-listed limitations.

Despite these limitations, our findings, in conjunction with the recent study by Verspeek & Stevens [30], suggest that bonobos may be sensitive to unfair treatment as per the IA responses known in humans. Such a shared trait has implications for evolutionary hypotheses, as it may reflect either the preservation of an ancestral mechanism for promoting cooperation in the hominoid and hominin lineages or the independent evolution of this behaviour in response to similar social pressures. Given that IA seems to be absent in the other *Pan* species (chimpanzees) [25,31], the results speak to convergent evolution rather than evolutionary preservation, in which case it would be logical to assume its presence as well in the chimpanzee. Furthermore, as bonobos are a highly cooperative species [27,28], our findings are consistent with the idea that IA evolved as a mechanism to stabilize and maximize the payoffs of cooperation, although this would be true for chimpanzees as well [40,58]. More research is needed to resolve this matter, perhaps with a wider range of observational approaches (e.g. [59]), and experimental tests in the context of cooperative exchanges between the two interactors rather than with a third partner.

## Data Availability

All data used in this study are available at SURFdrive [60]. Supplementary material is available online [61].

## References

[1] Rilling JK, Gutman DA, Zeh TR, Pagnoni G, Berns GS, Kilts CD. 2002 A neural basis for social cooperation. Neuron **35**, 395–405. (10.1016/s0896-6273(02)00755-9) 12160756

[2] Clutton-Brock T. 2009 Cooperation between non-kin in animal societies. Nature **462**, 51–57. (10.1038/nature08366) 19890322

[3] Fehr E, Fischbacher U. 2003 The nature of human altruism. Nature **425**, 785–791. (10.1038/nature02043) 14574401

[4] Raihani NJ, McAuliffe K. 2012 Does inequity aversion motivate punishment? Cleaner fish as a model system. Soc. Justice Res. **25**, 213–231. (10.1007/s11211-012-0157-8)

[5] Henrich J *et al*. 2005 'Economic man' in cross-cultural perspective: behavioral experiments in 15 small-scale societies. Behav. Brain Sci. **28**, 795–815. (10.1017/s0140525x05000142) 16372952

[6] Brosnan SF. 2011 A hypothesis of the co-evolution of cooperation and responses to inequity. Front. Neurosci. **5**, 43. (10.3389/fnins.2011.00043) 21519380 PMC3077916

[7] Geraci A, Surian L. 2011 The developmental roots of fairness: infants’ reactions to equal and unequal distributions of resources. Dev. Sci. **14**, 1012–1020. (10.1111/j.1467-7687.2011.01048.x) 21884317

[8] Blake PR *et al*. 2015 The ontogeny of fairness in seven societies. Nature **528**, 258–261. (10.1038/nature15703) 26580018

[9] Clark MS, Grote NK. 2003 Close relationships. In Handbook of psychology: personality and social psychology, vol. 5, pp. 447–461. Hoboken, NJ: John Wiley & Sons.

[10] Wu Y, Leliveld MC, Zhou X. 2011 Social distance modulates recipient’s fairness consideration in the dictator game: an ERP study. Biol. Psychol. **88**, 253–262. (10.1016/j.biopsycho.2011.08.009) 21889968

[11] Brosnan SF, de Waal FBM. 2003 Monkeys reject unequal pay. Nature **425**, 297–299. (10.1038/nature01963) 13679918

[12] Wynne CDL. 2004 Fair refusal by capuchin monkeys. Nature **428**, 140–140. (10.1038/428140a) 15014490

[13] Dubreuil D, Gentile MS, Visalberghi E. 2006 Are capuchin monkeys (Cebus apella) inequity averse? Proc. R. Soc. B **273**, 1223–1228. (10.1098/rspb.2005.3433) PMC1560285 16720395

[14] Bräuer J, Call J, Tomasello M. 2006 Are apes really inequity averse? Proc. R. Soc. B **273**, 3123–3128. (10.1098/rspb.2006.3693) PMC1679898 17015338

[15] Roma PG, Silberberg A, Ruggiero AM, Suomi SJ. 2006 Capuchin monkeys, inequity aversion, and the frustration effect. J. Comp. Psychol. **120**, 67–73. (10.1037/0735-7036.120.1.67) 16551166

[16] Sheskin M, Ashayeri K, Skerry A, Santos LR. 2014 Capuchin monkeys (Cebus apella) fail to show inequality aversion in a no-cost situation. Evol. Hum. Behav. **35**, 80–88. (10.1016/j.evolhumbehav.2013.10.004)

[17] McAuliffe K, Chang LW, Leimgruber KL, Spaulding R, Blake PR, Santos LR. 2015 Capuchin monkeys, Cebus apella, show no evidence for inequity aversion in a costly choice task. Anim. Behav. **103**, 65–74. (10.1016/j.anbehav.2015.02.014)

[18] Brosnan SF, Talbot C, Ahlgren M, Lambeth SP, Schapiro SJ. 2010 Mechanisms underlying responses to inequitable outcomes in chimpanzees, Pan troglodytes. Anim. Behav. **79**, 1229–1237. (10.1016/j.anbehav.2010.02.019) 27011389 PMC4801319

[19] van Wolkenten M, Brosnan SF, de Waal FBM. 2007 Inequity responses of monkeys modified by effort. Proc. Natl Acad. Sci. USA **104**, 18854–18859. (10.1073/pnas.0707182104) 18000045 PMC2141866

[20] Bräuer J, Call J, Tomasello M. 2009 Are apes inequity averse? New data on the token‐exchange paradigm. Am. J. Primatol. **71**, 175–181. (10.1002/ajp.20639) 19021260

[21] McGetrick J, Range F. 2018 Inequity aversion in dogs: a review. Learn. Behav. **46**, 479–500. (10.3758/s13420-018-0338-x) 30105647 PMC6268111

[22] Brosnan SF, de Waal FBM. 2014 Evolution of responses to (un)fairness. Science **346**, 1251776. (10.1126/science.1251776) 25324394 PMC4451566

[23] Massen JJM, Van Den Berg LM, Spruijt BM, Sterck EHM. 2012 Inequity aversion in relation to effort and relationship quality in long‐tailed macaques (Macaca fascicularis). Am. J. Primatol. **74**, 145–156. (10.1002/ajp.21014) 22038902

[24] Bräuer J, Hanus D. 2012 Fairness in non-human primates? Soc. Justice Res **25**, 256–276. (10.1007/s11211-012-0159-6)

[25] Ritov O, Völter C, Raihani N, Engelmann J. 2023 Are nonhuman animals averse to inequity? A meta-analysis. PsyArXiv. See https://psyarxiv.com/86vkf/ (accessed 31 January 2023).

[26] Prüfer K *et al*. 2012 The bonobo genome compared with the chimpanzee and human genomes. Nature **486**, 527–531. (10.1038/nature11128) 22722832 PMC3498939

[27] Furuichi T. 2019 Bonobo and chimpanzee: the lessons of social coexistence. Singapore: Springer.

[28] Samuni L, Surbeck M. 2023 Cooperation across social borders in bonobos. Science **382**, 805–809. (10.1126/science.adg0844) 37972165

[29] Brosnan SF. 2006 Nonhuman species’ reactions to inequity and their implications for fairness. Soc. Justice Res. **19**, 153–185. (10.1007/pl00022136)

[30] Verspeek J, Stevens JMG. 2023 Behavioral and physiological response to inequity in bonobos (Pan paniscus). Am. J. Primatol. **85**, e23455. (10.1002/ajp.23455) 36419405

[31] Engelmann JM, Clift JB, Herrmann E, Tomasello M. 2017 Social disappointment explains chimpanzees’ behaviour in the inequity aversion task. Proc. R. Soc. B **284**, 20171502. (10.1098/rspb.2017.1502) PMC5577499 28835562

[32] Titchener R, Thiriau C, Hüser T, Scherberger H, Fischer J, Keupp S. 2021 Social disappointment and partner presence affect long-tailed macaque refusal behaviour in an ‘inequity aversion’ experiment. PsyArXiv. See https://psyarxiv.com/bn4dz/. 10.1098/rsos.221225 PMC9974291 36866079

[33] Brosnan SF, Schiff HC, de Waal FBM. 2005 Tolerance for inequity may increase with social closeness in chimpanzees. Proc. R. Soc. B **272**, 253–258. (10.1098/rspb.2004.2947) PMC1634968 15705549

[34] Martin P, Bateson PPG, Bateson P. 1993 Measuring behaviour: an introductory guide. Cambridge, UK: Cambridge University Press.

[35] R Core Team. 2020 R: a language and environment for statistical computing. Vienna, Austria: R Foundation for Statistical Computing.

[36] Bates D, Mächler M, Bolker B, Walker S. 2014 Fitting linear mixed-effects models using lme4. J. Stat. Softw **67**, 1–48. (10.18637/jss.v067.i01)

[37] Forstmeier W, Schielzeth H. 2011 Cryptic multiple hypotheses testing in linear models: overestimated effect sizes and the winner’s curse. Behav. Ecol. Sociobiol. **65**, 47–55. (10.1007/s00265-010-1038-5) 21297852 PMC3015194

[38] Lenth R *et al*. 2020 Emmeans: estimated marginal means, aka Least-Squares Means. R Package Version 1.4.6.. See https://cran.r-project.org/web//packages/emmeans/emmeans.pdf.

[39] Barr DJ, Levy R, Scheepers C, Tily HJ. 2013 Random effects structure for confirmatory hypothesis testing: keep it maximal. J. Mem. Lang. **68**, 255–278. (10.1016/j.jml.2012.11.001) PMC3881361 24403724

[40] Hare B, Melis AP, Woods V, Hastings S, Wrangham R. 2007 Tolerance allows bonobos to outperform chimpanzees on a cooperative task. Curr. Biol. **17**, 619–623. (10.1016/j.cub.2007.02.040) 17346970

[41] Nolte S, Sterck EHM, van Leeuwen EJC. 2023 Does tolerance allow bonobos to outperform chimpanzees on a cooperative task? A conceptual replication of Hare et al., 2007. R. Soc. Open Sci. **10**, 220194. (10.1098/rsos.220194) 36686553 PMC9810421

[42] Hopper LM, Lambeth SP, Schapiro SJ, Brosnan SF. 2014 Social comparison mediates chimpanzees’ responses to loss, not frustration. Anim. Cogn. **17**, 1303–1311. (10.1007/s10071-014-0765-9) 24880642 PMC4676562

[43] Brosnan SF, Bshary R. 2016 On potential links between inequity aversion and the structure of interactions for the evolution of cooperation. Behaviour **153**, 1267–1292. (10.1163/1568539x-00003355)

[44] Mitani JC. 2009 Cooperation and competition in chimpanzees: current understanding and future challenges. Evol. Anthropol **18**, 215–227. (10.1002/evan.20229)

[45] Stanford CB. 2018 The new chimpanzee: a twenty-first-century portrait of our closest kin. Cambridge, MA: Harvard University Press.

[46] Mustoe AC, Harnisch AM, Hochfelder B, Cavanaugh J, French JA. 2016 Inequity aversion strategies between marmosets are influenced by partner familiarity and sex but not by oxytocin. Anim. Behav. **114**, 69–79. (10.1016/j.anbehav.2016.01.025) 27019514 PMC4802974

[47] Lehmann J, Ross C. 2011 Baboon (Papio anubis) social complexity—a network approach. Am. J. Primatol. **73**, 775–789. (10.1002/ajp.20967) 21563205

[48] Range F, Leitner K, Virányi Z. 2012 The influence of the relationship and motivation on inequity aversion in dogs. Soc. Justice Res. **25**, 170–194. (10.1007/s11211-012-0155-x)

[49] Brosnan SF, Hopper LM, Richey S, Freeman HD, Talbot CF, Gosling SD, Lambeth SP, Schapiro SJ. 2015 Personality influences responses to inequity and contrast in chimpanzees. Anim. Behav. **101**, 75–87. (10.1016/j.anbehav.2014.12.019) 25722495 PMC4337034

[50] Kret ME, Jaasma L, Bionda T, Wijnen JG. 2016 Bonobos (Pan paniscus) show an attentional bias toward conspecifics’ emotions. Proc. Natl Acad. Sci. USA **113**, 3761–3766. (10.1073/pnas.1522060113) 26976586 PMC4833271

[51] van Berlo E, Bionda T, Kret ME. 2023 Attention toward emotions is modulated by familiarity with the expressor: a comparison between bonobos and humans. Emotion **23**, 1904–1917. (10.1037/emo0000882) 36595387

[52] Tan J, Hare B. 2013 Bonobos share with strangers. PLoS One **8**, e51922. (10.1371/journal.pone.0051922) 23300956 PMC3534679

[53] Amici F, Widdig A, von Fersen L, Lopez Caicoya A, Majolo B. 2021 Intra-specific variation in the social behavior of Barbary macaques (Macaca sylvanus). Front. Psychol. **12**, 666166. (10.3389/fpsyg.2021.666166) 34721132 PMC8548740

[54] Kaufhold SP, van Leeuwen EJC. 2019 Why intergroup variation matters for understanding behaviour. Biol. Lett. **15**, 20190695. (10.1098/rsbl.2019.0695) 31718514 PMC6892515

[55] Samuni L, Wegdell F, Surbeck M. 2020 Behavioural diversity of bonobo prey preference as a potential cultural trait. eLife **9**. (10.7554/elife.59191) PMC7462605 32869740

[56] Kaiser I, Jensen K, Call J, Tomasello M. 2012 Theft in an ultimatum game: chimpanzees and bonobos are insensitive to unfairness. Biol. Lett. **8**, 942–945. (10.1098/rsbl.2012.0519) 22896269 PMC3497113

[57] van Leeuwen EJC, Call J. 2017 Conservatism and ‘copy-if-better’ in chimpanzees (Pan troglodytes). Anim. Cogn. **20**, 575–579. (10.1007/s10071-016-1061-7) 27999955 PMC5394136

[58] Samuni L, Crockford C, Wittig RM. 2021 Group-level cooperation in chimpanzees is shaped by strong social ties. Nat. Commun. **12**, 539. (10.1038/s41467-020-20709-9) 33483482 PMC7822919

[59] Van Leeuwen EJC, Zimmermann E, Ross MD. 2011 Responding to inequities: gorillas try to maintain their competitive advantage during play fights. Biol. Lett. **7**, 39–42. (10.1098/rsbl.2010.0482) 20630892 PMC3030874

[60] Radovanović K, Lorskens A, Schütte S, Bräuer J, Call J, Haun DBM, van Leeuwen EJC. Data from: Bonobos respond aversively to unequal reward distributions, SURFdrive, Dataset https://surfdrive.surf.nl/files/index.php/s/ddLnSrCLbmssZOz 10.1098/rspb.2024.2873 PMC12001075 40237076

[61] Radovanovic K, Lorskens A, Schütte S, Bräuer J, Call J, Haun DBM *et al*. 2025 Supplementary material from: Bonobos respond aversively to unequal reward distributions. Figshare. (10.6084/m9.figshare.c.7763471) PMC12001075 40237076

